# Genetic mapping of *Uba3 ^O.2.2^* , a pupal lethal mutation in *Drosophila melanogaster*

**DOI:** 10.17912/micropub.biology.000542

**Published:** 2022-03-18

**Authors:** Elizabeth Mast, Kayla L Bieser, Mary Abraham-Villa, Vanessa Adams, Akinwonuola J Akinlehin, Lynarose Z Aquino, Joseph L Austin, Abigail K Austin, Carissa N Beckham, Ethan J Bengson, Amanda Bieszk, Brianna L Bogard, Rowan C Brennan, Rebecca M Brnot, Nicholas J Cirone, Mason R Clark, Brianna N Cooper, Dennys Cruz, Katlyn A Daprizio, Jason DeBoe, Michaela M Dencker, Laura L Donnelly, Leanne Driscoll, Ryan J DuBeau, Sirada W Durso, Adam Ejub, Waad Elgosbi, Melanie Estrada, Kaeli Evins, Pearl D Fox, Jacob M France, Maira G Franco Hernandez, Lizbeth A Garcia, Olivia Garl, Myeerah R Gorsuch, Mikayla A Gorzeman-mohr, Madison E Grothouse, Megan E Gubbels, Romina Hakemiamjad, Chloé V Harvey, Madeline A Hoeppner, Jessica L Ivanov, Veronica M Johnson, Jessica L Johnson, Ashton Johnson, Kaleigh Johnston, Katie R Keller, Breanna T Kennedy, Levi R Killian, Marissa Klumb, Olivia L Koehn, Aaron S Koym, Kari J Kress, Regan E Landis, Kaitlyn N Lewis, Enosh Lim, Ilcen K Lopez, D’Artagnan Lowe, Paula Luengo Carretero, Grace Lunaburg, Samantha L Mallinder, Natalie A Marshall, Jessica Mathew, Jasmine Mathew, Hailee S Mcmanaway, Emily N Meegan, Jacob D Meyst, Meredith J Miller, Colin K Minogue, Alina A Mohr, Cristhian I Moran, Adrian Moran, Morgan D Morris, Michael D Morrison, Emmily A Moses, Cade J Mullins, Citlalli I Neri, Jess M Nichols, Breanna R Nickels, Akosua M Okai, Chiedu Okonmah, Makena Paramo, Meagan Paramo, Sydney L Parker, Neil K Parmar, Jacob Paschal, Prem Patel, Deep Patel, Erica B Perkins, Madelyn M Perry, Zachary Perry, Amanda A Pollock, Oxxyris Portalatin, Kamron S Proffitt, Jason T Queen, Alexis C Quemeneur, Amelia G Richardson, Kaylee Rosenberger, Allison M Rutherford, Itchel X Santos-Perez, Christy Y Sarti, Lacey J Schouweiler, Lauren M Sessing, Sara O Setaro, Christopher F Silvestri, Olivia A Smith, Mackenzie J Smith, Jayson C Sumner, Rachel R Sutton, Lindsay Sweckard, Nicholas B Talbott, Peyton A Traxler, Jenna Truesdell, Aaron F Valenti, Leif Verace, Pragathi Vijayakumar, William L Wadley, Katherine E Walter, Ayanna R Williams, Trey J Wilson, Makayla A Witbeck, Trinity M Wobler, Lucas J Wright, Karolina A Zuczkowska, Olivier Devergne, Danielle R Hamill, Hemin P Shah, Jamie Siders, Elizabeth E Taylor, Alysia D Vrailas-Mortimer, Jacob D Kagey

**Affiliations:** 1 Nevada State College; 2 Northern Illinois University; 3 Ohio Northern University; 4 Ohio Wesleyan University; 5 Illinois State University; 6 University of Detroit Mercy

## Abstract

An EMS mutagenesis screen was conducted in *Drosophila melanogaster* to identify growth control mutants. The multi-institution Fly-CURE consortium phenotypically characterized the *O.2.2* mutant using the *FLP/FRT* system which displayed a mutant lethal phenotype with reduced head development, and darkened ocular tissue. Complementation mapping was conducted to identify the affected gene. A failure to complement was identified in *Uba3* , resulting in the identification of the novel allele, *Uba3 ^O.2.2^ . Uba3* is a known disruptor of the cell cycle and our data are consistent with early larval/embryonic lethality displayed in numerous species.

**Figure 1. Characterization of the lethal Uba3 O.2.2 mutation by phenotypic analysis and complementation mapping.**
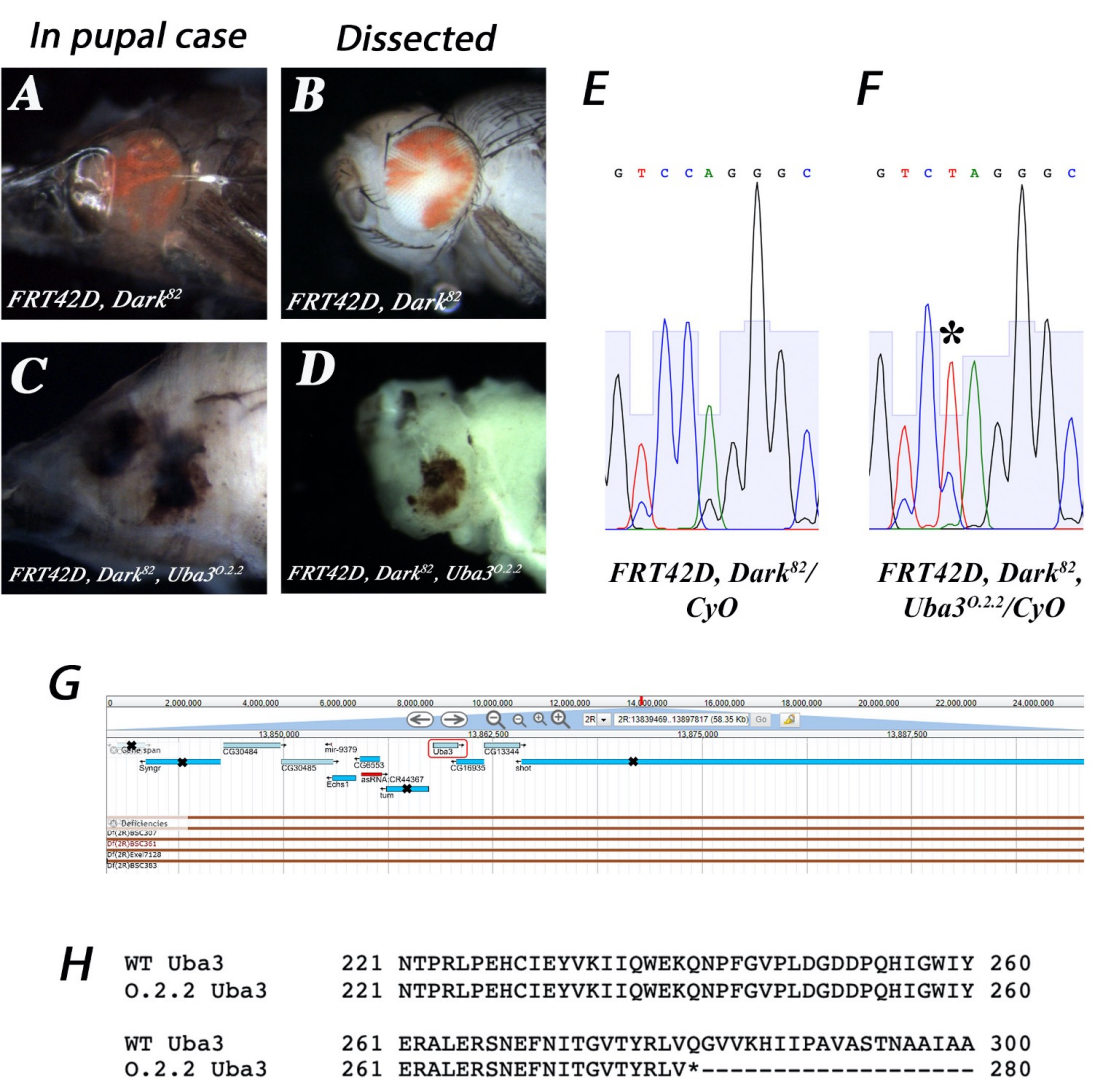
*
FRT42D, Dark ^82^* control (A-B) display a mosaic eye of red (mw ^+^ ) (mutant) to white (mw ^-^ ) (wildtype) tissue in a viable pupa. (C-D) *FRT42D, Dark ^82^ , Uba3 ^O.2.2^* mutant was pupal lethal, displaying a reduced head and darkened ocular tissue. (E) Sanger sequencing analysis of *FRT42D, Dark ^82^* control and (F) *FRT42D, Dark ^82^ , Uba3 ^O.2.2^* mutant indicating a heterozygous peak at 2R:13,859,838 (C to T). (G) The smallest region the *O.2.2* mutant failed to complement was 2R:13,839,479..13,897,827 as defined by the leftmost breakpoint of Df(2R)BSC383 and the rightmost breakpoint of Df(2R)Exel7128. All genes with an “X” were tested and complemented *O.2.2* with only the *Uba3 ^SH2028^* failing to complement. (H) Amino acid alignment of *FRT42D, Dark ^82^* control (WT *Uba3* ) to *FRT42D, Dark ^82^ , Uba3 ^O.2.2^* mutant ( *O.2.2 Uba3* ) displaying the nonsense mutation in *Uba3 ^O,2,2^ .* Image adapted from flybase.org (Gramates et al., 2017).

## Description

Induced ethyl methanesulfonate (EMS) mutations were genetically screened utilizing the FLP/FRT recombinase system to study cell overgrowth phenotypes in mosaic eyes of *Drosophila melanogaster* . EMS is a mutagen typically resulting in single nucleotide substitutions. Homozygosity was induced in the eye utilizing the FLP/FRT mitotic recombination system to study phenotype developments without homozygous lethality throughout the remainder of the specimen. It has been previously reported that genes impacting cell growth or division (such as *Dark, Ptc* ) trigger apoptosis leading to cell death (Akdemir *et al.* 2006). In the presence of *Dark ^82^* , apoptosis is blocked, and the overgrowth phenotype persists in an observable manner (Kagey *et al.* 2012). Male stock *Drosophila* (genotype *FRT42D, Dark ^82^ , O.2.2/CyO* and genotype *FRT42D, Dark ^82^ /CyO* ) were crossed with females (genotype *FRT42D; Ey-Flp* ) to promote mitotic recombination, creating offspring exhibiting either the control mosaic eye phenotype or the mutant phenotype (Figures 1A-D). The control cross (Figures 1A and 1B) displayed a typical phenotypic red:white ratio in pharate adults. The *O.2.2* mutant, however, was pupal lethal, displaying an underdeveloped head and black pigmented eye tissue (Figures 1C and 1D).

To identify the gene locus responsible for the *O.2.2* mutation, complementation mapping was conducted by undergraduate researchers at Nevada State College, Northern Illinois University, Albion College, Ohio Northern University, and Ohio Wesleyan University participating in the Fly-CURE consortium. This mapping was conducted during the COVID-19 pandemic which resulted in data collection through in-person, hybrid, and virtual courses. Complementation mapping has been successfully utilized by undergraduates to map the location of mutations driving the phenotypes produced from the FLP/FRT screen (Talley *et al.* 2021). Virgin female *Drosophila* (genotype *FRT42D, Dark82, O.2.2/CyO* ) were crossed with males from each of the 86 deficiency stocks from the Bloomington Stock Center 2R Deficiency Kit (that are distal to the *FRT42D* site). As the *O.2.2* mutation is homozygous lethal, F1 offspring were scored for the presence or absence of straight wings with the absence of straight wings an indicator of a failure to complement (Cook *et al.* 2012) (Table 1). The initial round of mapping resulted in three deficiency stocks failing to complement: *Df(2R)CX1* , *Df(2R)BSC383* , and *Df(2R)BSC307* . Two additional stocks outside of the 2R kit were tested resulting in the smallest region of failure to complement *O.2.2* of 2R:13,839,479..13,897,827 (Figure 1E). Students then selected seven genes within this region for further study (Table 1). Alleles of six of these genes complemented the *O.2.2* mutation (Table 1). We tested two alleles of *Uba3* : *Uba3 ^G8197^* and *Uba3 ^SH2028^ ,* which are both homozygous lethal transgenic insertion mutations and *Uba3 ^SH2028^* has previously been identified as a null mutation (Du *et al.* 2011). We found that the *Uba3 ^G8197^* allele complemented the O.2.2 mutation while the *Uba3 ^SH2028^* mutation failed to complement. We further investigated the *Uba3 ^G8197^* stock and found that it was no longer homozygous lethal, suggesting that this stock had lost the mutation in *Uba3* . The data we provided to the BDSC resulted in the removal of the *Uba3 ^G8197^* stock. As the *Uba3 ^SH2028^* allele failed to complement *O.2.2* (Table 1), students designed primers to different regions of the Uba3 gene and identified a nonsense mutation at 2R:13,859,838 (Gln281Stop at amino acid 281) leading to a premature stop codon.

Based upon the observed phenotype and genetic mapping in *Drosophila melanogaster* , we conclude that *O.2.2* is a novel allele of *Uba3* ( *Uba3 ^O.2.2^* ), resulting in a mosaic pupal lethal phenotype. *Uba3* encodes for a ubiquitin-like activating enzyme that adds NEDD8 (neural precursor cell expressed, developmentally downregulated 8; E1 ^NEDD8^ ) to proteins in a process called neddylation. Neddylation and the ubiquitin-proteasome system (UPS), which regulate proteolysis in the cell (Du *et al.* 2011, Nalepa *et al.* 2006), are critical for regulation of many developmental processes including multiple pathways for cell cycle progression (Tateishi *et al.* 2001, Nalepa *et al.* 2006). Evidence of *Uba3* knockdown in *Drosophila* and mice result in early larval/embryonic lethality (Du *et al.* 2011, Tateishi *et al.* 2001). The nonsense mutation we report is predicted to eliminate the E2-binding domain (associated with amino acids 354-443), which is a necessary conjugating enzyme in the NEED8 cascade (Huang *et al.* 2005). The elimination of this critical domain is consistent with the lethal phenotype observed in *Uba3 ^O.2.2^* .

Due to its role in disrupting the cell cycle, *Uba3* is a plausible early target for disrupting the cell cycle of the E1 activating enzyme in cancer cells. An inhibitor of E1 ^NEDD8^ , MLN4924, has been utilized *in vitro* to target cancer cells but its effectiveness was reduced in cells with *Uba3* mutations leading to resistance of MLN4924 (Xu *et al.* 2014). More recently, MLN4924, has shown promise in clinical trials for inhibiting growth and migration of cancer cells but a greater mechanistic understanding of the role of Uba3 in neddylation is still required as there appears to be an interplay between high glucose levels and the upregulation of *Uba3* (Du *et al.* 2021). Future characterizations in *Drosophila* may help to elucidate these complex relationships.

## Reagents

*w ^-^ ; FRT42D, Dark ^82^ /CyO* (Akdemir et al., 2006)

*w ^-^ ; FRT42D Dark ^82^ , Uba ^3O.2.2^ /CyO* (this study)

*w ^-^ ; FRT42D; Ey-Flp* (BDSC 8211)

Bloomington *Drosophila* Stock Center 2R Deficiency Kit *(Cook et al., 2012)*

*Additional Bloomington Stocks (See Table 1 for complete list of stock numbers)*
